# p53 status of newly established acute myeloid leukaemia cell lines

**DOI:** 10.1038/sj.bjc.6690064

**Published:** 1999-02

**Authors:** A Zheng, K Castren, M Säily, E-R Savolainen, P Koistinen, K Vähäkangas

**Affiliations:** Department of Clinical Chemistry, University of Oulu, Kajaanintie 50, Oulu, FIN-90220, Finland; Department of Internal Medicine, University of Oulu, Kajaanintie 50, Oulu, FIN-90220, Finland; Department of Pharmacology and Toxicology, University of Oulu, Kajaanintie 50, Oulu, FIN-90220, Finland

**Keywords:** p53, AML, cell lines

## Abstract

We analysed the status of the p53 gene and protein in eight newly established acute myeloid leukaemia (AML) cell lines representing blast cells of either de novo leukaemia patients in first remission or patients with relapsed and chemotherapy-resistant disease causing their death. There were no mutations in the p53 gene in any of the cell lines as analysed by single-strand conformation polymorphism of amplified exons 5–8. However, the p53 protein was clearly and consistently expressed in all of these cell lines, as shown by immunohistochemistry, Western blotting and flow cytometry. The consistently expressed p53 protein was located in both the nucleus and the cytoplasm of all the cell lines and, as shown by flow cytometry, it was mostly in a conformation typical of the mutated protein. These AML cell lines offer a tool for studying the production and function of the p53 protein and its possible role in cell cycle regulation and chemoresistance as well as in the regulation of apoptosis in AML. © 1999 Cancer Research Campaign


*British Journal of Cancer* (1999) **79**(3/4), 407–415
© 1999 Cancer Research Campaign
Article no. bjoc.1998.0064

### p53 status of newly established acute myeloid leukaemia cell lines

**A Zheng[1,2], K Castren[3], M Säily[1,2], E-R Savolainen[1], P Koistinen[2] and K Vähäkangas[3]**

Departments of [1]Clinical Chemistry, [2]Internal Medicine and [3]Pharmacology and Toxicology, University of Oulu, Kajaanintie 50, FIN-90220 Oulu, Finland

**Summary** We analysed the status of the p53 gene and protein in eight newly established acute myeloid leukaemia (AML) cell lines representing blast cells of either de novo leukaemia patients in first remission or patients with relapsed and chemotherapy-resistant disease causing their death. There were no mutations in the p53 gene in any of the cell lines as analysed by single-strand conformation polymorphism of amplified exons 5–8. However, the p53 protein was clearly and consistently expressed in all of these cell lines, as shown by immunohistochemistry, Western blotting and flow cytometry. The consistently expressed p53 protein was located in both the nucleus and the cytoplasm of all the cell lines and, as shown by flow cytometry, it was mostly in a conformation typical of the mutated protein. These AML cell lines offer a tool for studying the production and function of the p53 protein and its possible role in cell cycle regulation and chemoresistance as well as in the regulation of apoptosis in AML.

**Keywords:** p53; AML; cell lines

The expression of wild-type (wt) p53 protein is usually undetectable but, when the cells are exposed to physical or chemical genotoxic stimuli, the levels rise rapidly, leading to either arrest in the cell cycle or initiation of apoptosis (Kastan et al, 1991; Fritsche et al, 1993; Lu and Lane, 1993; Tishler et al, 1993).

In cancer, the function of p53 is often disturbed (for reviews, see Selivanova and Wiman, 1995; Gottlieb and Oren, 1996; Hainaut and Vähäkangas, 1997). In a large proportion of human solid tumours, missense mutations can be found to occur in the area of the central DNA-binding domain of p53 (reviewed by Greenblatt et al, 1994; Hollstein et al, 1994; Levine, 1997). Mutations often cause changes in the conformation of the protein and lead to inactivation of p53 (Zhang et al, 1992; Milner, 1994; Hainaut, 1995). The conformation of p53 may, however, also be changed for reasons other than missense mutations, such as changes in the redox condition, temperature and phosphorylation status of the protein (Hainaut, 1995; Steegenga, 1996; Levine, 1997). Binding to products of many viral oncogenes as well as the cellular oncoprotein mdm-2 may inactivate the p53 protein (Linzer and Levine, 1979; Bargonetti et al, 1991; Momand et al, 1992). Mutation or binding often leads to an increased half-life of p53 and in such cases, even without any evidence of growth arrest or apoptosis, the expression of p53 protein is easily observable in cells (for reviews see, for example, Gottlieb and Oren, 1996; Harris, 1996; Ko and Prives, 1996).

Because the inactivation of p53 in cancer has been associated with poor survival, refractory disease and chemoresistance (Lowe et al, 1993; Soini et al, 1993; Marks et al, 1996; Dive 1997), it is conceivable that restoring the function of p53 in cancer cells is worth development in cancer treatment (for a review see e.g. Harris 1996). The first in vivo studies on p53 gene therapy in lung cancer have already been published (Fujiwara et al, 1994; Roth et al, 1996). A plausible new form of cancer therapy would be to activate p53 by restoring the wt p53 conformation of cancer cells (Harris, 1996; Levine, 1997). This would become possible through characterization of the p53 regulatory pathways.

Relapses and chemoresistance continue to be major problems in malignant haematological disorders, especially in AML, which causes death in over 80% of patients over 65 years old (Hamblin, 1995). However, mutations in the p53 gene have been found to be very rare in these disorders (Greenblatt et al, 1994). In AML, the incidence of mutations is as low as less than 5% (Fenaux et al, 1992; Schottelius et al, 1994). One possible explanation for this could be that AML is a disorder in which p53 is inactive for reasons other than mutations.

This paper reports characterization of the p53 status of eight new AML cell lines established at the Oulu University Hospital. The cell lines were derived from patients who either are still in first remission (>3 years) or who died from a chemoresistant and relapsed disease. We show that p53 is consistently expressed at high levels in all the cell lines without mutations in exons 5–8. If the p53 protein is inactive in these cell lines, as is probable, the restoration of such conformation-based inactivation of p53 would be very interesting in terms of the development of leukaemia treatment.

### MATERIALS AND METHODS

### Source of cells

The study was carried out in accordance with the Helsinki Declaration and approved by the Ethics Committee of the Medical Faculty of University of Oulu. After obtaining informed consent, peripheral blood samples for the study were drawn at the same time as those for clinical tests before chemotherapy. The AML blast cells that gave rise to the cell lines were obtained from AML patients admitted to the Leukaemia Treatment Unit at the Department of Internal Medicine, University Hospital of Oulu.

*Received 12 February 1998*
*Revised 17 April 1998*
*Accepted 6 May 1998*

*Correspondence to:* P Koistinen

The diagnosis of AML was based on May–Grünwald–Giemsa (MGG), Sudan black and esterase stainings of bone marrow and blood smears according to the French–American–British (FAB) classification criteria (Bennet et al, 1976). The clinical features of the patients and the corresponding nomenclature of the cell lines are given in Tables 1 and 2. The patients numbered 1, 2, 3 and 8 represent de novo AML and those numbered 4–7 represent relapsed and clinically chemoresistant disease. The chemotherapy of the patients was carried out by following the AML-86 treatment protocol of the Finnish Leukaemia Group (Elonen, 1993).

### Establishment of cell lines

Blast cells were separated from peripheral blood by Ficoll–metrizoate (Nycomed, Oslo, Norway) density-gradient centrifugation. After isolation, the cells were cryopreserved at –70°C in the presence of 50% heat-inactivated fetal calf serum (FCS, Gibco, Grand Island, NY, USA), 10% dimethyl sulphoxide (Aldrich-Chemie, Steinheim, Germany) and α-minimal essential medium (α-MEM; Gibco). For the cell cultures, the blast cells were quickly thawed, washed twice with α-MEM and cultured at a high cell density of 1–2 × $10^6$ $ml^{-1}$ in α-MEM and 10% FCS in the presence of the following growth factors: 100 U $ml^{-1}$ interleukin -3 (IL-3) and IL-6 (Sandoz, Forschungsinstitut, Vienna, Austria), 100 U $ml^{-1}$ granulocyte–macrophage colony-stimulating factor (Novartis, Helsinki, Finland) and 40 ng $ml^{-1}$ mast cell growth factor (Immunex Corporation, Seattle, WA, USA). After culturing for 6–12 weeks in the above-mentioned culture medium, the cells were allowed to proliferate in 10% FCS and α-MEM for the next 3 months. The cells were then frozen at –70°C. Before p53 analysis, the cell lines were grown in the presence of 10% FCS and α-MEM for more than 2 years. The cells were cultured in suspensions in a moist atmosphere at 37°C with 5% carbon dioxide. The medium was changed every 3–4 days during the experiments. The doubling time of the cell line cells varied from 2.5 to 3.5 days, the mean value being 2.9 ± 0.3 days.

The cell lines were numbered from 1 to 8 for the corresponding patients presented in Table 1, and they were labelled as OU (Oulu University)–AML cell lines.

### Controlling for mycoplasma contamination

To detect possible mycoplasma in the cell cultures, a nucleic acid hybridization based technique was used (Gen-Probe Mycoplasma T.C. rapid detection system, Gen-Probe, San Diego, CA, USA). Until now, all the cell lines have proved to be negative for mycoplasma.

### Morphology, immunophenotype and karyotype analysis of cell lines

The morphology of the cell line cells was estimated from MGG-stained-cytospin preparations. Immunophenotype analyses were carried out by using monoclonal antibodies against differentiation antigens and a FACSort flow cytometer (Becton & Dickinson). The informative immunophenotypes are presented in Table 2 (corresponding available immunophenotype of native peripheral blood (PB) samples are given in parentheses). None of the cell lines expressed glycophorin A antigen or following cluster and differentiation antigens (CD): CD3, CD19, CD61 or CD117. Typical morphology pattern of the cells is shown in Figure 1. Cell line karyotypes were analysed by a standard Giemsa banding technique. The karyotype was diploid in the cell lines 2 and 3 and hyperdiploid (triploid or tetraploid) in the others. Otherwise, the chromosome patterns were chaotic, containing innumerable changes. However, no structural changes were observed in the chromosome 17p in any of the cell lines.

### Analysis of p53 mutations with single-strand conformation polymorphism (SSCP)

Exons 5–8 of the p53 gene were amplified individually by PCR using two sets of intron primers for each of the exons, the second set being internal to the first one (nested primers) (Lehman et al, 1991). The primers were kindly provided by Dr Curtis C Harris (Laboratory of Human Carcinogenesis, NIH, NCI, Bethesda, MD 20892, USA). Dynazyme DNA polymerase and the corresponding buffer (Finnzymes, Espoo, Finland) were used in the polymerase chain reaction (PCR) with other reagents and under the reaction conditions described previously (Vähäkangas et al, 1992).

**Table 1** Clinical data of the patients

| Patient | Age/sex (years) | FAB | Response to initial therapy | Outcome (months from diagnosis)[a] | Disease status[b] |
|---|---|---|---|---|---|
| AML-1 | 26/F | M4 | CR (C+BMT) | CR (50+) | Diagnosis |
| AML-2 | 52/F | M2 | CR (C) | CR (60+) | Diagnosis |
| AML-3 | 48/M | M4 | CR (C) | CR (66+) | Diagnosis |
| AML-4 | 39/M | M2 | CR (C+BMT) | Death in first relapse (7) | First relapse |
| AML-5 | 70/M | M5 | CR (C) | Death in second relapse (28) | Second relapse |
| AML-6 | 47/F | M1 | CR (C) | MDS, death in first leukemic relapse (62) | First relapse |
| AML-7 | 63/F | M4 | CR (C) | Death in first relapse (14) | First relapse |
| AML-8 | 63/F | M4 | CR (C) | CR (43+) | Diagnosis |

[a]Status in April 1998. [b]Status at sample collection for cell culture. F, female; M, male; FAB, French–American–British classification of acute myeloid leukaemia; CR, complete remission; C, chemotherapy; BMT, bone marrow transplantation; MDS, myelodysplastic syndrome.

 © Cancer Research Campaign 1999

**Table 2** Immunophenotype of the OU–AML cell lines. Corresponding values of native patient samples are presented in parentheses

| | Antigen-positive cells (%) | | | | | |
|---|---|---|---|---|---|---|
| **Cell line** | **CD34** | **CD33** | **CD13** | **CD14** | **CD7** | **MPO** |
| OU–AML-1 | 0 (11) | 97 (69) | 93 (31) | 0 (34) | 0 (0) | 0 |
| OU–AML-2 | 0 (0) | 100 (75) | 87 (76) | 0 (1) | 0 (16) | 0 |
| OU–AML-3 | 0 (0) | 95 (85) | 76 (55) | 0 (44) | 69 (10) | 0 |
| OU–AML-4 | 0 (34) | 97 (93) | 94 (79) | 0 (0) | 25 (21) | 0 |
| OU–AML-5 | 0 | 96 | 98 | 0 | 28 | 0 |
| OU–AML-6 | 0 | 83 | 67 | 0 | 67 | 0 |
| OU–AML-7 | 0 (23) | 100 (97) | 93 (46) | 1 (10) | 62 (12) | 0 |
| OU–AML-8 | 0 (3) | 98 (97) | 89 (77) | 0 (69) | 19 (16) | 0 |

CD, cluster of differentiation, MPO, myeloperoxidase.

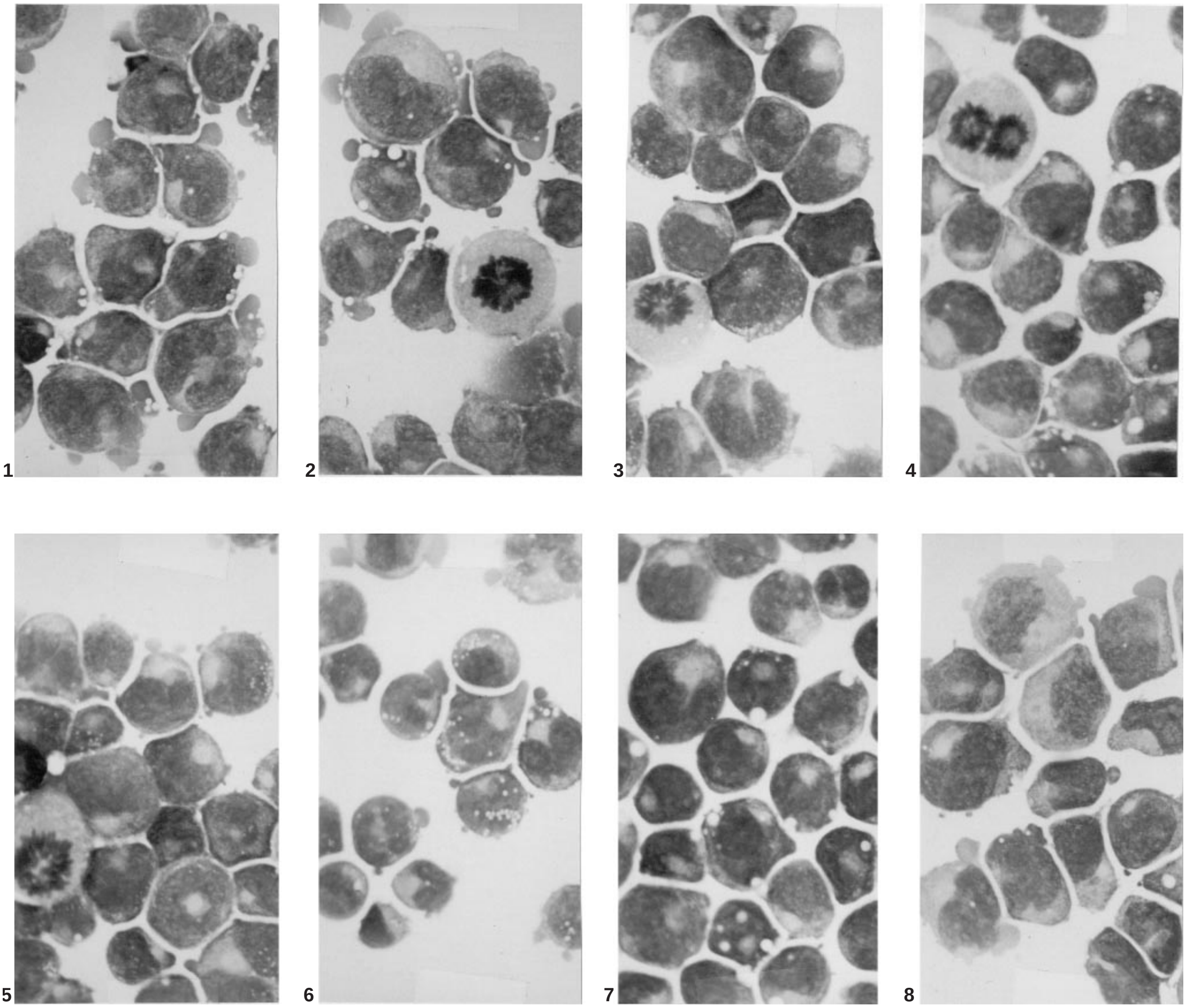

**Figure 1** Morphology of OU–AML cell lines 1–8. Magnification × 500

Negative controls (reaction mixture without the template) were included in each amplification in order to test for contaminations. The size of the amplified DNA was controlled electrophoretically in a 3% NuSieve 3:1 agarose gel (FMC, Finnzymes) with molecular size markers. The amplified DNA was purified by running in a 3% NuSieve 3:1 agarose gel. The bands of appropriate sizes

*© Cancer Research Campaign 1999*

were cut out of the gel and the DNA was eluted from the gel slices with ammonium acetate. Eluted DNA was precipitated with 100% ethanol in a freezer and the precipitated DNA was dissolved into 30–50 µl of TE buffer.

For SSCP, a 1:1 mixture of the purified DNA and a bromophenol blue-formaldehyde stop solution (Sequenase Kit, US Biochemicals) was denatured for 5 min at 100°C, and 1 µl of the mixture was used for each run (Welsh et al, 1997). The samples were loaded on a 20% homogeneous polyacrylamide gel, and the gel was run by Pharmacia Phastsystem semi-dry electrophoresis equipment with neutral buffer strips (Pharmacia Biotech, Uppsala, Sweden). The gels were stained with the silver staining kit (Pharmacia) according to the manufacturer's instructions. For negative controls, p53 exons were amplified from wt lymphocyte DNA. For a positive control, lymphocyte DNA was amplified using a mutated 5′ primer (Welsh et al, 1997). We have shown that the efficiency in detecting mutations within p53 exons 5–8 of our SSCP method is 98%, i.e. 98% of known mutations can be detected (Welsh et al, 1997).

### p53 protein immunohistochemistry

The cells were prepared for immunohistochemistry as described previously (Rämet et al, 1995) by fixing in 10% neutral formalin for 2–3 days at room temperature, after which the cells were pelleted by centrifugation. The cell pellet was suspended in melted 2% agarose, and the agarose block was further embedded in paraffin. Four-micron-thick sections were placed on slides and stained for p53 protein by using the routine avidin–biotin–peroxidase staining method. The primary antibody was polyclonal rabbit anti-human CM-1 antibody (Novocastra Laboratories, Newcastle upon Tyne, UK), which detects both the wild-type and the mutated p53 protein. At least 500 p53-positive cells were counted per sample. The experiments were done in triplicate.

### Western blotting

The cells harvested from the suspension cultures were first washed twice with phosphate-buffered saline (PBS) and then lysed in two ways as follows: for whole-cell extracts, $2 \times 10^6$ cells were directly lysed in 50 µl of Laemmli sample buffer, and for nuclear and cytoplasmic extracts, $1 \times 10^7$ cells were lysed in 150 µl of a low-salt Hepes buffer (20 mM Hepes, 20% glycerol, 10 mM sodium chloride, 1.5 mM magnesium chloride, 0.2 mM EDTA, 1 mM dithiothreitol, 0.1% NP40). Both buffers contained the following protease inhibitors: 500 mM phenylmethylsulphonyl fluoride, 2 mg $ml^{-1}$ aprotinin, 1.4 mg $ml^{-1}$ pepstatin A and 1 mg $ml^{-1}$ antipain. After 10 min on ice, the lysed cells were centrifuged at 2000 r.p.m. for 4 min and the supernatant (containing the whole-cell or cytoplasmic extract) was collected. The pellet from low-salt Hepes buffer was lysed further in 50 µl of Hepes buffer with a high salt content of 500 mM sodium chloride. The second supernatant (containing the nuclear extract) was collected after shaking the mixture at +4°C and centrifugation at 14 000 r.p.m. for 15 min. The protein contents of the cytoplasmic and nuclear extracts were determined by Bio-Rad protein assay (Bio-Rad Laboratories, USA). Cell lysate (15 µl) and 20 µg of cytoplasmic or 40 µg of nuclear extract were applied to 12% SDS-PAGE (Bio-Rad, Hercules, CA, USA) and transferred on to Hypond nitrocellulose membrane (Amersham Life Science, Buckinghamshire, UK). After blocking for 30 min with 8% dried fat-free milk in Tris-buffered saline–Tween (TBS-T) (20 mM Tris-HCl pH 7.6, 137 mM sodium chloride, 0.1% Tween-20), the membranes were incubated for 1 h with the primary antibody, a mouse anti-human p53 DO7 (Novocastra Laboratories Ltd, Newcastle Upon Tyne, UK), diluted in the blocking solution at 1:10 000. The specific p53 protein–antibody complex was detected by using a secondary antibody, horseradish peroxidase-conjugated sheep anti-mouse immunoglobulin (Amersham) and an enhanced chemiluminescence (ECL) detection kit (Amersham). OVCAR-3 ovarian carcinoma cell line cells, which express both cytoplasmic and nuclear p53 protein (Rämet et al 1998), were used in all experiments as positive controls. p53 protein is as a denatured form in Western blot analysis. DO7 antibody will recognize both mutated and wt protein. Molecular weight markers ascertained that studied p53 protein was 53 kDa.

### Flow cytometry

When analysed by flow cytometry, the p53 protein keeps its native, non-denatured form (Zhu et al, 1993). To study the protein conformation of native p53, the cell lines were investigated flow cytometrically by using three different monoclonal anti-p53 antibodies. For the analyses, the cells were harvested from the suspension cultures, washed twice with PBS, and then treated with 70% cold ethanol for 15 min and washed twice with PBS. The permeabilized cells were incubated for 30 min at room temperature with one of the mouse anti-human p53 monoclonal antibodies or with a non-specific mouse isotype control. The antibody-treated cells were washed twice with PBS and incubated with fluorescein isothiocyanate (FITC)-conjugated rabbit anti-mouse $F(ab)_2$ fragments (Dakopatts, Glostrup, Denmark) for 30 min. The cells were then washed twice with PBS and a total of $10^4$ cells were analysed using a FACSort flow cytometer (Becton-Dickinson).

The following monoclonal mouse anti-human antibodies were used in the flow cytometer analyses: DO7, Ab3 (clone PAb 240) and Ab5 (clone PAb 1620), the first being purchased from Novocastra Lab and the others from Oncogene Research Products/Calbiochem (Cambridge, MA, USA). Ab5 recognizes only the wt p53 protein, whereas Ab3 detects only a mutated form of the p53 protein. The mutated p53 protein recognized by Ab3 can be either a protein translated from a mutated p53 gene or a wt p53 protein that is only in a mutational protein conformation (Zhu et al, 1993). The antibody DO7 recognizes the p53 protein regardless of the conformation.

### RESULTS

### SSCP analysis

No mutations in the p53 gene were found in any of the cell lines analysed by PCR-SSCP of amplified exons 5–8 (Figure 2). As shown by Welsh et al (1997), the clearly visible differences in the band patterns of the positive controls confirmed the success of the analysis by SSCP.

### Immunohistochemistry

Each cell line cultured for over 2 years was studied by immunohistochemistry. Expression of p53 was consistently observed in each of the cell lines, although the number of positive cells per cell line varied from one experiment to the next (Table 3).

 © Cancer Research Campaign 1999

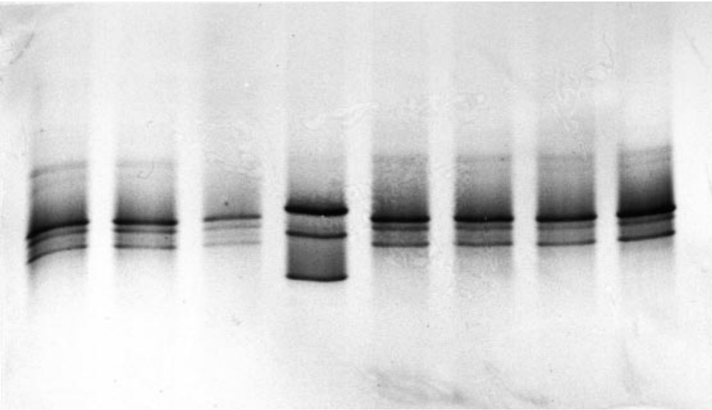

**Figure 2** An example of single-strand conformation polymorphism (SSCP). p53 exon 7 was amplified using intron primers, gel purified and subjected to SSCP (for details see Materials and methods). 1, OU–AML-1; 2, OU–AML-2; 3, negative control (p53 exon 7 amplified using lymphocyte DNA as a template); 4, positive control (p53 exon 7 amplified using lymphocyte DNA as a template and a 5′ primer with an inserted mutation); 5, OU–AML-3; 6, OU–AML-4; 7, OU–AML-5; 8, OU–AML-6

**Table 3** p53 positivity by immunohistochemistry. Percentage of p53-positive cells when analysed by polyclonal antibody CM-1. Mean ± s.d. of three experiments

| Cell line | Number of positive cells (%)[a] |
|---|---|
| OU–AML-1 | 14 ± 3 |
| OU–AML-2 | 13 ± 8 |
| OU–AML-3 | 25 ± 8 |
| OU–AML-4 | 33 ± 10 |
| OU–AML-5 | 29 ± 16 |
| OU–AML-6 | 20 ± 6 |
| OU–AML-7 | 17 ± 6 |
| OU–AML-8 | 16 ± 13 |

[a]Cells with nuclear and/or cytoplasmic staining were counted as positive. At least 500 cells were counted per preparation.

Figure 3 shows an example of the p53 staining pattern of the OU–AML cell line 4. An OVCAR-3 ovarian cancer cell line, shown previously to express an abundance of the p53 protein (Rämet et al, 1998), was used as a positive control for both nuclear and cytoplasmic p53 expression. In the cell lines, the expression of p53 was localized in both compartments of the cell.

### Western blotting

D07 antibody proved to be very sensitive in Western blotting and detected a single p53 band in whole-cell lysates as well as nuclear and cytoplasmic preparations in each of the cell lines studied. Every experiment was repeated three or four times. The representative results from one experiment are shown in Figure 4.

From three of the cell lines (4, 5 and 8) it was also possible to analyse the p53 levels in corresponding non-cultured native cells as well as in cells that had been cultured for only 6 weeks. Barely detectable amounts of p53 protein were seen in the native cells, while p53 expression was stronger, although minimal, in the cells from the 6-week-old cultures. The most pronounced p53 expression was always detected in the cell line cells (data not shown). The expression of p53 in each of the cell lines was constant; during a 4-day follow-up the level of p53 protein did not vary when the whole-cell lysate samples were analysed every 4–6 h (data not shown).

### Flow cytometry

Five of the OU-AML cell lines (3, 4, 5, 7 and 8) were analysed by flow cytometry. The analyses were repeated twice with each of the different anti-p53 antibodies. The proportions of positive cells in the samples (mean ± s.d.) are shown in Table 4. The results from two representative cell lines are shown as histograms (Figure 5). In each of the cell lines, p53 was expressed mostly in a mutational conformation, whereas a negligible number of cells contained p53 in the wt conformation.

### DISCUSSION

In the present study, the p53 gene and protein status in eight newly established AML cell lines was analysed. Although p53 was overexpressed in all of the cell lines, as shown by immunohistochemistry, Western blotting and flow cytometry, no mutations in the exons 5–8 of the gene were found, as analysed by PCR-SSCP. As the SSCP method that we used, utilizing, for example, two temperatures for each exon and a good temperature control, is able to detect 98% of mutations within p53 exons 5–8, few additional data would be expected by sequencing (Welsh et al, 1997).

There are altogether 11 exons in the p53 gene. Most of the p53 mutations in cancer cells occur in exons 5–8 (Greenblatt et al, 1994), and mutations in the other exons are very exceptional. As we did not find any mutations in this region of the p53 gene, it is probable that p53 mutations had played no role in the leukaemogenesis or the relapsed and chemoresistant disease of the patients who donated the cells. On the other hand, the overexpression of p53 in tumour cells implicates inactivation of the protein. The present work also showed that cell culture conditions per se do not

© Cancer Research Campaign 1999 

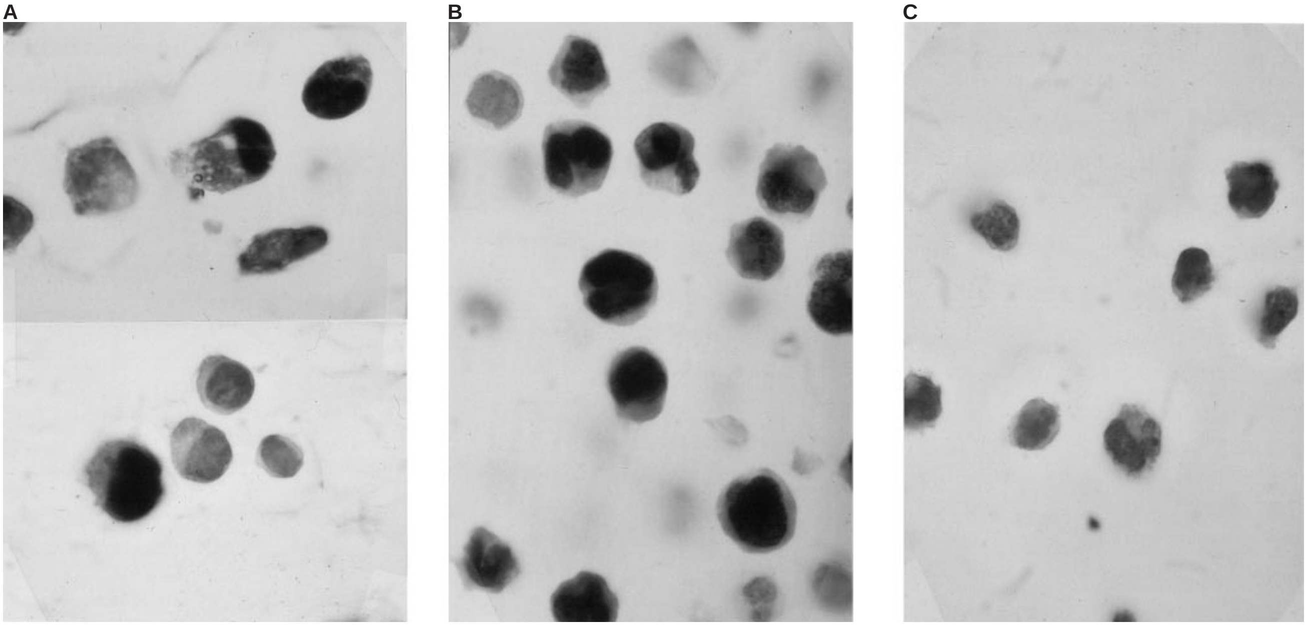

**Figure 3** p53 protein analysed by immunohistochemistry using the polyclonal antibody CM-1. (**A**) Positively stained OVCAR-3 ovarian cancer cell line cells used as controls. (**B**) Positively stained OU–AML-4 cell line cells. (**C**) Negatively stained OU–AML-4 cell line cells. Magnification × 500

predispose AML cells to p53 mutations, and mutations are not a requirement for the establishment of AML cell lines, as suggested earlier by Sugimoto et al (1992). They found mutations in the p53 gene in nine out of ten myeloid cell lines studied.

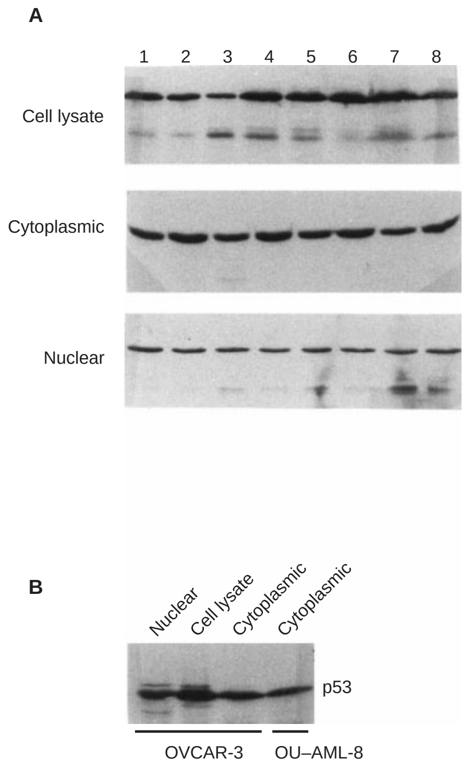

**Figure 4** Expression of p53 protein by Western blotting. (**A**), OU–AML cell lines 1–8. (**B**) OVCAR-3 cell line as a control. Cell lysate (15 μl) and 20 μg of cytoplasmic or 40 μg of nuclear extract/sample were loaded onto SDS polyacrylamide gel and visualized by enhanced chemiluminescence and DO7 antibody.* An unspecific band found in all Western blottings with monoclonal antibodies of class IgG immunoglobulins

It was previously assumed that if the p53 protein is detected in cancer cells it has to be a mutated protein. It was later recognized, however, that there are cases and tumour types that express high levels of p53 protein in the absence of mutations of the p53 gene (Peng et al, 1993; Castren et al, 1998; for review, see Hall and Lane, 1994). The present results show that AML also belongs to this category of malignancies.

Although immunoprecipitation would be the most sensitive method for the definition of p53 protein conformation, flow cytometry provides a good alternative. Ab3 anti-p53 antibody has been used in flow cytometric analysis (Zhu et al, 1993,1994; Bi et al, 1994). It recognizes both mutated p53 and wt protein in mutational, i.e. promoter but not in suppressor, conformation and also reacts with denatured wt protein (Gannon et al, 1990; Rivas et al, 1992; Zhang et al, 1992; Bi et al, 1994). On the other hand, Ab5 antibody, although not introduced for flow cytometry, is known to recognize p53 protein only in wt, i.e. suppressor, conformation, but not in promoter conformation (Milner and Medcalf, 1991). It does not react with denatured or mutated protein either. In our cell lines, most of the p53 protein was recognized by Ab3 antibody, i.e. it was in a mutational or promoter conformation. Apart from in AML cells (Rivas et al, 1992; Zhang et al, 1992; Zhu et al, 1993, 1994), the mutational p53 conformation has also been detected in

**Table 4** p53 positivity as determined by flow cytometry. Percentage of p53 positive cells when analysed by monoclonal anti-p53 antibodies DO7, Ab3 and Ab5 (two analyses, mean ± s.d.)

| | Anti-p53 antibodies | | |
|---|---|---|---|
| **Cell line** | **Do7** | **Ab3** | **Ab5** |
| OU–AML-3 | 96 ± 4 | 89 ± 15 | 0 ± 0 |
| OU–AML-4 | 96 ± 4 | 85 ± 17 | 2 ± 2 |
| OU–AML-5 | 99 ± 2 | 83 ± 23 | 1 ± 1 |
| OU–AML-7 | 91 ± 10 | 77 ± 25 | 0 ± 0 |
| OU–AML-8 | 92 ± 6 | 66 ± 19 | 1 ± 1 |

 *© Cancer Research Campaign 1999*

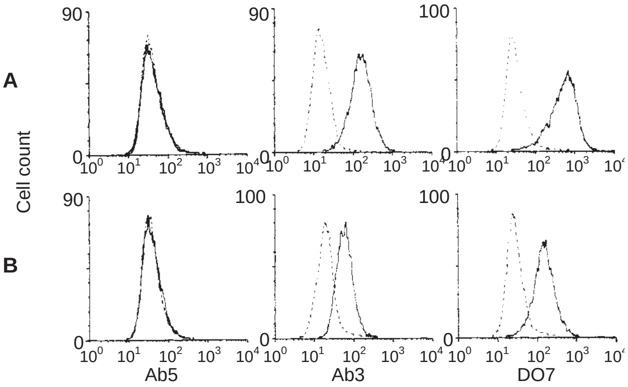

**Figure 5** The cell lines OU–AML-3 (**A**) and OU–AML-8 (**B**) were analysed by flow cytometry for cytoplasmic p53 protein expression by using three different monoclonal anti-p53 antibodies and FITC-conjugated $F(ab)_2$ fragments in a two-step staining method. The antibody DO7 detects the p53 protein both in the functional wild-type conformation as well as in the mutated conformation. Ab5 detects only the wild type and Ab3 the mutated conformation. The dotted histogram represents irrelevant isotype control normal haematopoietic progenitor cells (Rivas et al, 1992; Zhang et al, 1992; Bi et al, 1994). This conformation may represent a condition whereby wt p53 is in an inactive form and permits cell proliferation instead of acting as a suppressor of the cell cycle. Also, the fast growth of the cell line cells in suspension allows for the possibility that most of the p53 protein was not functional as a suppressor.

The immunohistochemistry and Western blotting analyses showed that the p53 protein was located both in the nucleus and in the cytoplasm of the cells in all the cell lines. In the literature, mainly nuclear localization of p53 has been reported. There are reports suggesting that cytoplasmic p53 represents an inactivated protein, which is logical in view of the fact that one of the main functions of the p53 protein is to act as a transcription factor (Funk et al, 1992). In these cell lines, p53 accumulation in cytoplasm may have been caused by a failure in the translocation of the protein to the nucleus (Moll et al, 1996). Alternatively, an unknown factor in the cytoplasm contributing to the mutated formation of the genetically wt p53 protein may have captured the p53 in the cytoplasm. We do not know what factor it is in the cell lines that changes the wt p53 protein conformation to a mutational one. In theory, it could be a protein inhibitor of some kind, such as a virus protein or mdm-2 (Linzer and Levine, 1979; Bargonetti et al, 1991; Momand et al, 1992). However, what we know for certain is that the inactivation is not due to proteolysis, which is known to occur in ubiquitin-mediated (Chowdary et al, 1994; Maki et al, 1996; Maki and Howley, 1997) and calpain systems (Kubbutat and Vousden, 1997; Zhang et al, 1997), because the protein detected by Western blotting was full sized, i.e. 53 kDa. Furthermore, no major changes in the p53 expression levels were observed in the repeated analyses performed on the cell lines within 72 h, which indicates well-balanced production and degradation of the p53 protein in the cells.

The p53 gene is located in the chromosome 17p13 (Isobe et al, 1986; Mcbride et al, 1986). In chromosomal analyses, the number of chromosomes in the cell lines was higher than normal, and several chromosomes 17 were frequently seen, which could partly explain the observed overexpression of the p53 protein in the cell lines.

p53 provides an attractive target for drug action because its inactivation has been shown to be related to disease progression and poor outcome in many types of cancer (Soini et al, 1993; Imamura et al, 1994; Wada et al, 1994; Döhner et al, 1995; Marks et al, 1996; Dive 1997). On the other hand, p53-dependent apoptosis is known to suppress tumour growth and progression in vivo (Symonds et al, 1994). When the wt p53 gene is transfected to cancer cells containing a mutated and inactivated p53 gene, it often stops the cell cycle and induces apoptosis in the cells (for a review see Levine, 1997). It has also been shown that p53-dependent apoptosis modulates the cytotoxic effects of both ionizing radiation and common anti-tumour agents, such as fluorouracil, etoposide and doxorubicin. Cells lacking wt p53 are resistant to these agents, whereas cells expressing wt p53 are sensitive to them and undergo cell death by apoptosis (Lowe et al, 1993; Chresta et al, 1996).

In AML, mutations of the p53 gene are rare (Fenaux et al, 1992; Schottelius et al, 1994). Based on the present findings and according to the previously published studies (Zhang et al, 1992; Zhu et al, 1993, 1994), it is probable that inactivation of the wt p53 protein in AML is due to a change in the protein conformation. In order to improve AML treatment, it would be plausible to try to develop drugs that are able to convert the inactive p53 protein conformation to an active one. Such a restored p53 activity might inhibit AML cell growth or increase the susceptibility of these cells to standard treatments. Changes in the p53 protein conformation have been achieved in embryonal carcinoma cells by exposing them to etoposide (Lutzker and Levine, 1996).

In conclusion, the p53 gene and protein status in eight newly established autonomously growing AML cell lines were analysed. All the cell lines overexpressed p53 protein, although there were no p53 mutations at the gene level. As measured by flow cytometry, a small part of the observed wt p53 protein was in a true wt conformation, while most was in a mutational conformation, which could mean that most of the p53 protein in the cell lines was not functional, as in its usual role as a suppressor of the cell cycle. Clarification of the mechanisms of p53 inactivation could lead to the possible to restoration of its normal function. This, as well as the possible consequences of p53 inactivity for the survival and proliferation status of AML cells, is currently being studied.

### ACKNOWLEDGEMENTS

This study was supported by the Finnish Cancer Societies, the Paulo Foundation and the Oulu University Hospital, Finland. We especially want to acknowledge Drs Paavo Pääkkö and Mika Rämet for helping with the immunohistochemistry technique and Dr Robert Vinqvist for analysing the karyotype of the cell lines. We also thank Professor Olavi Pelkonen for critical reading of the manuscript.

### REFERENCES

Bargonetti J, Friedman PN, Kern SE, Vogelstein B and Prives C (1991) Wild-type but not mutant p53 immunopurified proteins bind to sequences adjacent to the SV40 origin of replication. *Cell* **65**: 1083–1091

Bennett JM, Catovsky D, Daniel MT, Flandrin G, Galton OAG, Gralnick HD and Sultan C (1976) Proposals for the classification of acute leukemias. *Br J Haematol* **33**: 451–458

Bi S, Lanza F and Goldman JM (1994) The involvement of tumor suppressor p53 in normal and chronic myelogenous leukemia hemopoiesis. *Cancer Res* **54**: 582–586

Castren K, Vähäkangas K, Heikkinen E and Ranki A (1998) Absence of p53 mutations in benign and premalignant male genital lesions with overexpressed p53 protein. *Int J Cancer* **77**: 674–678

Chowdary DR, Dermody JJ, Jha KK and Ozer HL (1994) Accumulation of p53 in a mutant cell line defective in the ubiquitin pathway. *Mol Cell Biol* **14**: 1997–2003

Chresta CM, Masters JRW and Hickman JA (1996) Hypersensitivity of human testicular tumors to etoposide induced apoptosis is associated with functional p53 and a high bax:bcl-2 ratio. *Cancer Res* **56**: 1834–1841

Dive C (1997) Avoidance of apoptosis as a mechanism of drug resistance. *J Intern Med* **242** (suppl. 740): 139–145

Döhner H, Fischer K, Bentz M, Hansen K, Benner A, Cabot G, Diehl D, Schlenk R, Coy J, Stilgenbauer S, Volkmann M, Galle PR, Postka A, Hunstein W and Lichter P (1995) p53 gene deletion predicts for poor survival and non-response to therapy with purine analogs in chronic B-cell leukemias. *Blood* **85**: 1580–1589

Elonen E for the Finnish Leukemia Group (1993) Intensive chemotherapy of adult acute myelogenous leukemia: a randomized trial between 4 and 8 cycles. *Blood* **82** (suppl. 1): 328a

Fenaux P, Preudhomme C, Quiquandron I, Jonveaux P, Lai JL, Vanrumbeke M, Loucheux-Lefebvre MH, Bauters F, Berger R and Kerckaert JP (1992) Mutations of the p53 gene in acute myeloid leukemia. *Br J Haematol* **80**: 178–183

Fritsche M, Haessler C and Brandner G (1993) Induction of nuclear accumulation of the tumor-suppressor protein p53 by DNA-damaging agents. *Oncogene* **8**: 307–318

Fujiwara T, Grimm EA, Mukhopadhyay T, Zhang W-W, Owen-Schaub LB and Roth JA (1994) Induction of chemosensitivity in human lung cancer cells in vivo by adenovirus-mediated transfer of the wild-type p53 gene. *Cancer Res* **54**: 2287–2291

Funk WD, Pak DT, Karas RH, Wright WE and Shay JW (1992) A transcriptionally active DNA-binding site for human p53 protein complexes. *Mol Cell Biol* **12**: 2866–2871

Gannon JV, Greaves R, Iggo R and Lane DP (1990) Activating mutations in p53 produce a common conformational effect. A monoclonal antibody specific for the mutant form. *EMBO J* **9**: 1595–1602

Gottlieb TM and Oren M (1996) p53 in growth control and neoplasia. *Biochim Biophys Acta* **1285**: 77–102

Greenblatt MS, Bennet WP, Hollstein M and Harris CC (1994) Mutations in the p53 tumor suppressor gene: clues to cancer etiology and molecular pathogenesis. *Cancer Res* **54**: 4855–4878

Hainaut P (1995) The tumor suppressor protein p53: a receptor to genotoxic stress that controls cell growth and survival. *Curr Op Oncol* **7**: 76–82

Hainaut P and Vähäkangas K (1997) p53 as a sensor of carcinogenic exposures: mechanisms of p53 protein induction and lessons from p53 gene mutations. *Pathol Biol* **45**: 833–844

Hall PA and Lane DP (1994) p53 in tumor pathology: can we trust immunohistochemistry? – revisited! *J Pathol* **172**: 1–4

Hamblin TJ (1995) Disappointments in treating acute leukemia in the elderly. *N Engl J Med* **332**: 1712–1713

Harris CC (1996) Structure and function of the p53 tumor suppressor gene: clues for rational cancer therapeutic strategies. *J Natl Cancer Inst* **88**: 1442–1455

Hollstein M, Rice K, Greenblatt MS, Soussi T, Fuchs R, Sorlie T, Hoving E, Smith-Sorensen B, Montesano R and Harris CC (1994) Database of p53 gene somatic mutations in human tumors and cell lines. *Nucleic Acids Res* **22**: 3551–3555

Imamura J, Miyoushi I and Koeffler P (1994) p53 in hematological malignancies. *Blood* **84**: 2412–2421

Isobe M, Emanuel B, Gival D, Oren M and Croce C (1986) Localization of gene for human p53 tumor antigen to band 17p13. *Nature* **320**: 84–87

Kastan MB, Onyekwere O, Sidransky D, Vogelstein B and Craig RW (1991) Participation of p53 protein in the cellular response to DNA damage. *Cancer Res* **51**: 6304–6311

Ko LJ and Prives C (1996) p53: puzzle and paradigm. *Genes Dev* **10**: 1054–1072

Kubbutat MHG and Vousden KH (1997) Proteolytic cleavage of human p53 by calpain: a potential regulator of protein stability. *Mol Cell Biol* **17**: 460–468

Lehman TA, Bennett WP, Metcalf RA, Welsh JA, Ecker J, Modali RV, Ullrich S, Romano JW, Appella E, Testa JR, Gerwin BI and Harris CC (1991) p53 mutations, ras mutations, and p53 heat shock 70 protein complexes in human lung carcinoma cell lines. *Cancer Res* **51**: 4090–4096

Levine AJ (1997) p53, the cellular gatekeeper for growth and division. *Cell* **88**: 323–331

Linzer DI and Levine AJ (1979) Characterization of a 54K dalton cellular SV40 tumor antigen present in SV40-transformed cells and uninfected embryonal carcinoma cells. *Cell* **17**: 43–52

Lowe SW, Ruley HE, Jacks T and Houseman DE (1993) p53 dependent apoptosis modulates the cytotoxicity of anticancer cells. *Cell* **74**: 957–968

Lu X and Lane DP (1993) Differential induction of transcriptionally active p53 following UV or ionizing irradiation: defects in chromosome instability syndromes? *Cell* **75**: 765–778

Lutzker S and Levine AJ (1996) A functionally inactive p53 protein in embryonal carcinoma cells is activated by DNA damage or cellular differentiation. *Nature Med* **2**: 804–810

Maki CG and Howley PM (1997) Ubiquitination of p53 and p21 is differentially affected by ionizing and UV radiation. *Mol Cell Biol* **17**: 355–363

Maki CG, Huibregtse JM and Howley PM (1996) In vivo ubiquitination and proteasome-mediated degradation of p53. *Cancer Res* **56**: 2649–2654

Marks DI, Kurz BW, Link MP, Ng E, Shuster JJ, Lauer SJ, Brodsky I and Haines DS (1996) High incidence of potential p53 inactivation in poor outcome childhood acute lymphoblastic leukemia at diagnosis. *Blood* **87**: 1155–1161

Mcbride OW, Merry D and Givol D (1986) The gene for human p53 cellular tumor antigen is located on chromosome 17 short arm (17p13). *Proc Natl Acad Sci USA* **83**: 130–134

Milner J (1994) Forms and functions of p53. *Semin Cancer Biol* **5**: 211–219

Milner J and Medcalf EA (1991) Co-translation of activated mutant p53 with wild type drives the wild-type p53 protein into the mutant conformation. *Cell* **65**: 765–774

Moll OM, Ostermeyer AG, Halady R, Winkfield B, Frazier M and Zambetti G (1996) Cytoplasmic sequestration of wild-type p53 protein impairs the G1 checkpoint after DNA damage. *Mol Cell Biol* **16**: 1126–1137

Momand J, Zambetti GP, Olson DC, George D and Levine AJ (1992) The mdm-2 oncogene product forms a complex with the p53 protein and inhibits p53 mediated transactivation. *Cell* **69**: 1237–1245

Peng HQ, Hogg D, Malkin D, Bailey D, Gallie BL, Bulbul M, Jewett M, Buchanan J and Gross PE (1993) Mutations of the p53 gene do not occur in testis cancer. *Cancer Res* **53**: 3574–3578

Rämet M, Castren K, Järvinen K, Pekkala K, Tupeenniemi-Hujanen T, Soini Y, Pääkkö P and Vähäkangas K (1995) p53 protein expression is correlated with benzo[a]pyrene-DNA adducts in carcinoma cell lines. *Carcinogenesis* **16**: 2117–2124

Rämet M, Turunen S, Pääkkö P, Laatio L, Raunio H, Hainaut P and Vähäkangas K (1998) Benzo(a)pyrene induces p53 and apoptosis but no G1 arrest in MCF-7 cells. (submitted)

Rivas CI, Wisniewski D, Strife A, Perez A, Lambek C, Bruno S, Darzynkiewicz Z and Clarkson B (1992) Constitutive expression of p53 protein in enriched normal human marrow blast cell populations. *Blood* **79**: 1982–1986

Roth JA, Nguyen D, Lawrence DD, Kemp BL, Carrasco CH, Ferson DZ, Hong WK, Komaki R, Lee JJ, Nesbitt JC, Pisters KMW, Putnam JB, Schea R, Shin DM, Walsh GL, Dolormente MM, Han C-i, Martin FD, Yen N, Xu K, Stephens LC, McDonnel TJ, Mukhopadhyay T and Cai D (1996) Retrovirus-mediated wild-type p53 gene transfer to tumors of patients with lung cancer. *Nature Med* **2**: 985–991

Schottelius A, Brennscheidt U, Ludwig W-D, Mertelsmann RH, Herrmann F and Lübbert M (1994) Mechanisms of p53 alteration in acute leukemias. *Leukemia* **8**: 1673–1681

Selivanova G and Wiman KG (1995) p53: a cell cycle regulator activated by DNA damage. *Adv Cancer Res* **66**: 143–180

Soini Y, Turpeenniemi-Hujanen T, Kamel D, Autio-Harmainen H, Risteli J, Risteli L, Nuorva K, Pääkkö P and Vähäkangas K (1993) p53 immunohistochemistry in transitional cell carcinoma and dysplasia of the urinary bladder correlates with disease progression. *Br J Cancer* **68**: 1029–1035

Steegenga WT, van der Eb AJ and Jochemsen AG (1996) How phosphorylation regulates the activity of p53. *J Mol Biol* **263**: 103–113

Sugimoto K, Toyoshima H, Sakai R, Miyagawa K, Hagiwara K, Ishikawa F, Takaku F, Yazaki Y and Hirai H (1992) Frequent mutations in the p53 gene in human myeloid leukemia cell lines. *Blood* **79**: 2378–2383

Symonds H, Krall L, Remington L, Saenz-Robies M, Lowe S, Jacks T and Van Dyke T (1994) p53-dependent apoptosis suppresses tumor growth and progression in vivo. *Cell* **78**: 703–711

Tishler RB, Calderwood SK, Coleman N and Price BD (1993) Increases in sequence specific DNA binding by p53 following treatment with chemotherapeutic and DNA damaging agents. *Cancer Res* **53**: 2212–2216

Vähäkangas KH, Samet JM, Metcalf RA, Welsh JA, Bennett WP, Lane DP and Harris CC (1992) Mutations of p53 and ras genes in radon-associated lung cancer from uranium miners. *Lancet* **339**: 576–580

Wada H, Asada M, Nakazawa S, Itoh H, Kobayashi Y, Inoue T, Fukumoro K, Chan L, Sugita K, Hanada R, Akuta N, Kobayashi N and Mizutani S (1994) Clonal expansion of p53 mutant cells in leukemia progression in vitro. *Leukemia* **8**: 53–59

 *© Cancer Research Campaign 1999*

Welsh JA, Castren K and Vähäkangas K (1997) Single-strand conformation polymorphism analysis to detect p53 mutations: characterization and development of controls. *Clin Chem* **43**: 2251–2255

Zhang W, Hu G, Estey E, Hester J and Deisseroth A (1992) Altered conformation of the p53 protein in myeloid leukemia cells and mitogen-stimulated normal blood cells. *Oncogene* **7**: 1645–1647

Zhang W, Lu Q, Xie Z-J and Mellgren RL (1997) Inhibition of the growth of WI-38 fibroblasts by benzyloxycarbonyl-leu-leu-tyr diazomethyl ketone: evidence that cleavage of p53 by a calpain-like protease is necessary for G1 to S-phase transition. *Oncogene* **14**: 255–263

Zhu Y-M, Bradbury D and Russell N (1993) Expression of different conformations of p53 in the blast cells of acute myeloblastic leukaemia is related to in vitro growth characteristics. *Br J Cancer* **68**: 851–855

Zhu Y-M, Bradbury D and Russell N (1994) Wild-type p53 is required for apoptosis induced by growth factor deprivation in factor-dependent leukaemic cells. *Br J Cancer* **69**: 468–47

*© Cancer Research Campaign 1999*
